# Prescription Department

**Published:** 1893-09

**Authors:** 


DYSMENORRHOEA.

Dr. Thomas E. Craig, of Lawrenceburg, Ind., gives the following formula to The Prescription, containing 1-240 grain of corrosive sublimate to the dose :—

℞. Resinæ guaiacum,
Terebinth, Canadensis, aa ℥ j.
Oil sassafras .......... ʒ ij.
Alcohol ................ ℥ viij.
Hydrag. corros. chlor ....gr. ij.

Dissolve the guaiacum and Canada balsam in one-half of the alcohol, and the corrosive sublimate in the other half. Mix and filter, and then add the oil of sassafras.

Dose, 10 to 15 drops three times a day, to be given in a capsule or dropped on dry sugar.

INDOLENT BOWELS IN AGED PERSONS.

℞. Pulv. scammon, ...... ʒ ss.

Ext. aloes............ ʒ j.

Bals. Peru.............gr. x.

Ol. carui. .............m x.

M. et in pil. no. xx.

Sig : One at night—Dr. Robinson.

To allay the irritable cough in phthisis the following possesses considerable merit :

℞. Acidi hydrocyanici diluti, ʒ i.
Syrupi pruni Virginianæ, ℥ iii.
Morphinæ sulphatis, gr. ii.
Sig.—Teaspoonful every two or three hours.—Steer.

ECZEMA OF CHILDREN.

℞. Bismuth subnitr., 10.0,
Zinci. oxyd., 2.0.
Glycerini, 8.0.
Acid carbol., gtt xx.
Vaselin. albi, 30.0.

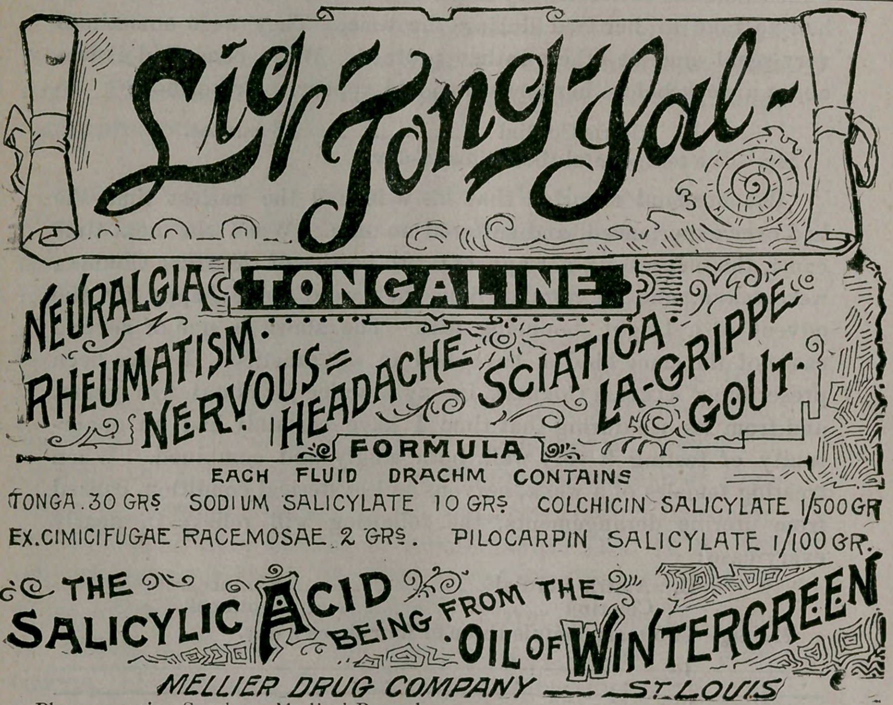

Please mention Southern Medical Record.

WHOOPING COUGH.

The Journal de Medicine de Paris advises the following spray from an atomizer in whooping cough :

℞. Acidi Carbolici (puræ) 2 grains.
Sodii Biboracis...... 1 drachm.
Sodii Bicarbonatis... 1 drachm.
Glycerini ........... 2 ounces.
Aquæ Destillatæ, q. s. 2 ounces.

M. et ft. solutio. Signa. Use as spray.

GRANULAR LIDS.

Dr. G. Sterling Ryerson orders, applied at night :

℞. Hydrarg. oxid. flav ....gr. iv.
Zinci oxid ............gr. ij.
Thymol ...............mij.
Camphor. .............gr. ss.
Cocain, muriat ........gr. ij.
Vaselin ..............℥j—M.

*Therap. Gazette.*

FOR COUGH.

℞. Antikamnia............ʒi

Salol.

Quin. Sulph......aa grs. xx

Spts Frumenti. ........℥iii

Syr. Tolutan ..........℥i

Syr. Simplex......q. s..℥vi

M. Sig.—One teaspoonful every hour until cough is relieved.

For the very painful affections like epididymitis, Prof. Steer recommends a formula like the following :

℞. Morph. sulph..........gr. i

Chloral hydrat..........ʒ iss

Potas. bromid..........ʒ ii

Syrup .................ʒ iv

Aquæ .................℥ iiss

M. Sig.—Tablespoonful every three hours, if necessary.

